# Hypoxia in Head and Neck Tumors: Characteristics and Development during Therapy

**DOI:** 10.3389/fonc.2013.00223

**Published:** 2013-08-28

**Authors:** Martin-Immanuel Bittner, Anca-Ligia Grosu

**Affiliations:** ^1^Department of Radiation Oncology, University Medical Center Freiburg, Freiburg, Germany

**Keywords:** tumor hypoxia, head and neck tumors, primary chemoradiotherapy, biological imaging, PET

## Abstract

Cancers of the head and neck are a malignancy causing a considerable health burden. In head and neck cancer patients, tumor hypoxia has been shown to be an important predictor of response to therapy and outcome. Several imaging modalities can be used to determine the amount and localization of tumor hypoxia. Especially PET has been used in a number of studies analyzing this phenomenon. However, only few studies have reported the characteristics and development during (chemoradio-) therapy. Yet, the characterization of tumor hypoxia in the course of treatment is of great clinical importance. Successful delineation of hypoxic subvolumes could make an inclusion into radiation treatment planning feasible, where dose painting is hypothesized to improve the tumor control probability. So far, hypoxic subvolumes have been shown to undergo changes during therapy; in most cases, a reduction in tumor hypoxia can be seen, but there are also differing observations. In addition, the hypoxic subvolumes have mostly been described as geographically rather stable. However, studies specifically addressing these issues are needed to provide more data regarding these initial findings and the hypotheses connected with them.

## Introduction: Relevance of Tumor Hypoxia

Cancers of the head and neck are a frequent malignancy, causing a high burden of disease with a median 5 year survival of around 50% (1). Tumor hypoxia has been shown to be a negative prognostic factor for cancers of the head and neck, being associated with reduced therapeutic effect of radiotherapy and decreased overall survival (2–7). This is explained by decreased sensitivity toward radiation and reduced accessibility for chemotherapy (1, 8). Therefore, tumor hypoxia is an important phenomenon in radiation oncology.

## Imaging Modalities

Novel imaging techniques have the potential to improve the therapeutic setting (9). 18-F-MISO-PET is probably the most commonly used and best validated tracer for hypoxia imaging so far (8). Gagel et al. (10) and Zimny et al. (11) found good correlations with pO_2_-polarography measurements in cancers of the head and neck. However, Mortensen et al. (12) were not able to confirm these results. It can be hypothesized that the different methods used for defining hypoxia all have their limitations (13).

Other PET tracers have also been proposed or used for hypoxia imaging in cancers of the head and neck, such as 18-F-FAZA (14, 15), 62-Cu-ATSM (16), 18-F-EF5 (17), or 18-F-HX4 (18). However, the superiority of certain tracers still remains to be elucidated. Another option is the use of dynamic PET imaging data, as proposed by several groups (19, 20). Interpreting the dynamic data can be challenging, given the fact that the models currently in use differ in the results obtained and conclusions drawn, as shown for a model comparison in a patient set (21). Further imaging modalities are amongst several others dynamic contrast enhanced MRI (22) or perfusion CT (23).

Most studies so far correlated one set of pre-treatment imaging data with the clinical outcome or another parameter of interest. This review however is dedicated to analyze the characteristics and development of hypoxic subvolumes during therapy, thus examining the results of serial imaging with special attention to possible implications for radiation treatment planning.

## Characteristics of Hypoxic Subvolumes and Development during Therapy

In the classical concept of tumor hypoxia, acute and chronic hypoxia can be differentiated. The former results from short-term perfusion changes whereas the latter is a consequence of a limited diffusion capacity of oxygen from the non-physiological tumor vessels (8). However, evidence regarding the changes of the hypoxic subvolumes during (chemoradio-) therapy remains scarce. Given the prognostic importance and the potential therapeutic consequences (e.g., alteration of radiotherapy, additional drugs), the analysis of tumor hypoxia has to be regarded as an important field of research.

Most studies report that during or after therapy residual or even increasing hypoxia is generally less frequent than decreasing or resolving hypoxia: Rischin et al. (5) found residual hypoxia in six out of 28 patients (pre-treatment vs. week 4 or 5), Lee et al. (24) found residual hypoxia in two out of 18 patients (pre-treatment vs. week 4) and Zips et al. (25) found residual hypoxia in 10 out of 24 patients (pre-treatment vs. week 5).

In contrast, higher proportions of residual hypoxia were found by Dirix et al. (26): four out of eight patients showed residual hypoxia (pre-treatment vs. week 4). In another study, residual hypoxia was detected in six out of 13 patients (pre-treatment vs. week 2–4) with hypoxic subvolumes at the same location, but smaller in size (27). Sometimes, however, hypoxic subvolumes can be so small that detailed reports regarding the behavior during therapy cannot be given; this was the case for a study including four patients with head and neck cancers, with hypoxic subvolumes between 0.0 and 2.7% (28).

In general, a remarkable reduction in tumor hypoxia in the course of treatment is a finding common to most studies performed so far.

**Figure 1:**
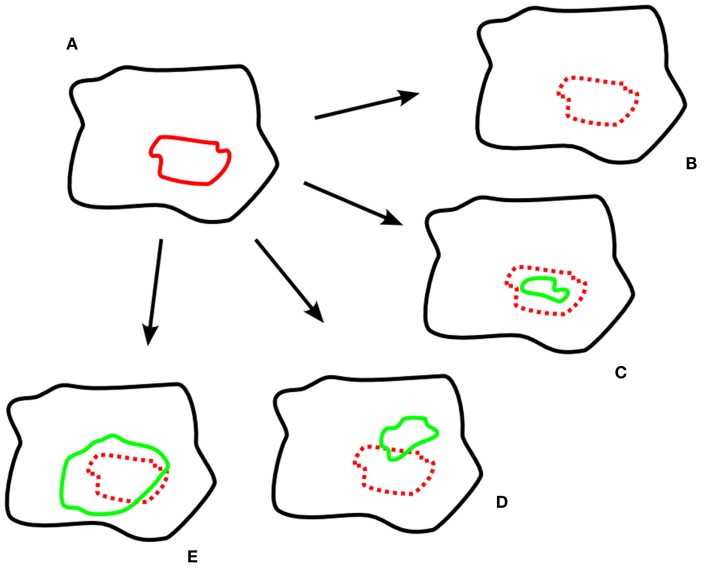
**Exemplary schematic hypoxia imaging scans**. **(A)** pre-treatment; **(B–E)** different developments during treatment; **(B)** resolved hypoxia; **(C)** decreased, geographically stable hypoxia; **(D)** decreased, geographically unstable hypoxia; **(E)** increased hypoxia. Legend: black: tumor volume; red: hypoxic subvolume pre-treatment; green: hypoxic subvolume during treatment.

As stated above, hypoxia remaining at the same location (but frequently decreasing in size) has been described before. Zips et al. (25) assessed the development of the hypoxic subvolumes in the course of chemoradiotherapy in four subsequent 18-F-MISO-PET-scans and also showed three different types of behavior: stable hypoxia, decreasing hypoxia, and increasing hypoxia (see Figure 1 for a schematic display of the different findings, also including other studies’ results).

A study dedicated at assessing the time-course of hypoxia during therapy has recently confirmed these types of behavior, at the same time adding new aspects: five out of 16 patients showed persistent hypoxia in serial 18-F-MISO-PET imaging and were subsequently analyzed in detail (29). In addition to the general types of behavior stated above, a stationary and a dynamic component were described. They refer to the degree of overlap of the hypoxic subvolumes in the course of treatment (pre-treatment vs. week 2). A stationary component was present in 4 of 5 and a dynamic component in 3 of 5 patients. These two labels are not mutually exclusive, since increasing hypoxia can also migrate into volumes which were not hypoxic before. Overall, a geographically rather stable conformation was found in the majority of patients (29).

The dynamic component has also been studied before: according to Lin et al. (30) and Nehmeh et al. (31), only 7 out of 13 patients showed a high (*R* ≥ 0.5) correlation between hypoxic subvolumes in two 18-F-MISO-PET-scans made 3 days apart, therefore excluding the effect of therapy. This has been interpreted as an indicator of on-going within-voxel changes of oxygenation status. Contrasting results have been obtained by Okamoto et al. (32), who found a high reproducibility of two 18-F-MISO-PET-scans made 2 days apart. However, simulation data indicate that acute hypoxia does not affect the reproducibility of 18-F-MISO-uptake in hypoxic subvolumes (33). Toma-Dasu et al. (34) proposed a mathematical algorithm based on a single 18-F-MISO-PET-scan prior to treatment to identify presumably more radioresistant subvolumes. This is clearly a field which needs further research.

Polarographic data also showed that oxygenation status/hypoxia can change in several ways in the course of treatment: Stadler et al. (35) found both increases and decreases of the hypoxic fraction. Similarly, dynamic imaging data also showed both decreasing and increasing hypoxia (36). Another way to analyze dynamic image data is by giving curves, representing accumulation, intermediate and wash-out types of hypoxia (37). In this study, a majority of patients (11 of 14) showed a decrease in tumor-muscle ratio. A clear qualitative decrease in tumor hypoxia over 4 weeks of treatment was also reported by another study (38).

It has also been reported that acute (changing) and chronic (stable) hypoxia can be modeled in serial 18-F-MISO-PET imaging using Gaussian and Poisson distributions (39). In this study, acute hypoxia accounted for an average amount of 34%. Again, this indicates a higher proportion of stable or chronic as opposed to changing tumor hypoxia.

Recently, it has been proposed that fluctuating hypoxia is also important because it may maintain cancer stem cells (40). This hypothesis clearly needs further confirmatory work, but it highlights the significance of tumor hypoxia for the outcome.

## Hypoxia Imaging in Other Entities

So far, most studies on hypoxia imaging have been conducted in patients with cancers of the head and neck. There are few data regarding other entities, and to our knowledge there are no data available for serial hypoxia imaging.

In uterine cervix carcinoma, 18-F-FETNIM-PET uptake was associated with a worse prognosis (41). However, for pancreatic cancer – known to be often hypoxic – results of a recent imaging study with 18-F-MISO-PET were not promising (42). Another small study with four heterogeneous gastrointestinal cancers, one lung cancer, and one uterine cancer showed no hypoxia for the lung cancer, a slight decrease in hypoxic subvolume for the uterine cancer (42.3–36.5%) and a mixed response for the gastrointestinal cancers: three relatively strong reductions and one slight increase (28).

In this regard, it will be interesting to see the results of an on-going exploratory study assessing serial 18-F-MISO-PET and serial functional MRI for non-small cell lung cancer (43).

## Clinical Implications

Given the findings discussed so far, it is important to consider possible implications not only for research, but also for the clinical setting. One way to address hypoxic subvolumes is through dose painting. This approach aims at delivering higher doses to potentially more radioresistant parts of a tumor. The possibility and feasibility of using hypoxia imaging in a clinical setting in head and neck cancer patients – e.g., as a template for dose painting – has already been shown (14, 44–47).

To-date, there are no clinical studies comparing the effects on response and outcome of an altered radiation regimen aiming at hypoxic subvolumes with a standard course of treatment.

Therefore, possible effects on the outcome can only be hypothesized; plan comparisons predict higher tumor control probabilities when delivering a 10-Gy simultaneous integrated boost to hypoxic subvolumes identified with a 18-F-MISO-PET-scan prior to treatment in an IMRT plan (48). An increased tumor control probability has also been calculated by Chang et al. (49), who compared a hypoxic subvolume-directed boost (total dose 84 Gy) with a standard plan (70 Gy) and a uniform dose escalation plan (84 Gy). In this study, the dose escalation to the hypoxic subvolume was found to be superior to the other plans. However, the clinical significance of these findings needs further investigation, ideally in a randomized controlled clinical trial. But there are also other aspects which have to be considered for radiation treatment planning, for example the number of fractions and fraction size (e.g., hypofractionation as one option with increased effectiveness) (50). Still, the radiobiology of radiation-induced changes in hypoxic subvolumes is not fully understood, with the effects of hypoxia on the outcome also depending on the tumor type and other characteristics (8).

On the other hand, based on the dynamic components of hypoxia, it has been questioned whether or not dose painting on potentially geographically unstable structures makes sense (51). There is no definite answer to this question yet, but a rather stable geographical location may advocate a possible inclusion into radiation treatment planning.

If hypoxia imaging is to be used in a clinical setting, the identification of the best time frame for image acquisition has also to be considered: on the one hand, most studies showed a substantial decrease of the amount of hypoxia in the course of treatment, with little or no residual hypoxia at the end of treatment (implying advantages of early imaging). On the other hand, at the beginning of treatment, a high proportion of patients seem to be partly hypoxic (implying advantages of later imaging). Taking into account these two possibly conflicting aspects, it has been suggested to use image data acquired after the first or second week of treatment [e.g., (25, 29)]. So far, this seems to be the most reliable timing for image acquisition.

Another possible intervention is the use of additional drugs or other modifiers specifically aiming at tumor hypoxia. Several studies have been performed, and a number of agents are currently under consideration. Especially nitroimidazoles, now known for their use as hypoxia-specific tracers, have been studied extensively due to their known hypoxia sensitizing effect (50). Other modifications include the inspiration of normobaric or hyperbaric oxygen, attempting to increase oxygen level and supply. In a recent meta-analysis, the positive effects of these interventions on locoregional control and overall survival – without thereby increasing the rate of complication – have been shown (50). Other targets include tumor vessels, e.g., by using VEGF, HIF-1alpha, or PI3K/Akt/mTOR inhibitors (52). Evidence for the use of these agents mostly comes from *in vitro* and (early) *in vivo* studies, and clinical evidence is rare. However, some drugs which have been in use for a long time for other indications revealed positive effects on tumor hypoxia and have recently been further studied in this regard. Prominent examples include HIV protease inhibitors such as Nelfinavir, currently studied in clinical trials (53).

## Conclusion

Hypoxic subvolumes in cancers of the head and neck are significantly associated with response to therapy and outcome. In the time-course of treatment, the characteristics of these subvolumes change. Few studies have addressed these changes so far. Major findings point toward a remarkable reduction in tumor hypoxia during treatment and a geographically rather stable conformation. However, more intense research is needed to further characterize the development of tumor hypoxia during treatment.

## Conflict of Interest Statement

The authors declare that the research was conducted in the absence of any commercial or financial relationships that could be construed as a potential conflict of interest.
